# Breast cancer awareness among women in the Syrian Coast: a cross-sectional study

**DOI:** 10.1097/MS9.0000000000000753

**Published:** 2023-04-27

**Authors:** Seif-Aldin Abdul Rahman, Haidara Kherbek, Sawsan Ismail, Ali Abdul Rahman, Jaafar Zahlout, Ibrahem Abboud, Munawar Hraib, Sarah Jouni, Tareq Turk, Yana Hleibieh, Khedr Layka, Sara Alaidi, Jana Skef, Somar Mansour, Michael Georgeos, Ousama Taweel, Zuheir Alshehabi

**Affiliations:** a Faculty of Medicine; b Department of Pathology, Cancer Research Center, Tishreen University; Departments of c Obstetrics & Gynecology; d Internal Medicine; e Oncology, Faculty of Medicine, Tishreen University; f Department of Statistics, Faculty of Economy, Tishreen University, Latakia, Syria

**Keywords:** Awareness, breast cancer, screening

## Abstract

Breast cancer is the most common malignancy in women and represents a leading cause of cancer-related mortalities. Females’ awareness toward breast cancer is essential for the early diagnosis and detection especially in low and middle-income countries. This study aims to assess the awareness of breast cancer risk factors among females in the Syrian Coast.

**Methodology:**

An online cross-sectional survey was conducted among Syrian females during the COVID-19 pandemic. The questionnaire used in the study was derived from the Cancer Research UK questionnaire and modified to fit the perspective of the study. Statistical Package for Social Sciences (SPSS) was used for data analysis. The independent samples *t*-test and the one-way analysis of variance (ANOVA) were applied to determine whether there is statistical evidence or any statistically significant differences between the variables.

**Results:**

A total of 1305 females participated in the study. The majority aged between 18 and 25, (28.8%) of the participants obtained their information related to breast cancer from internet, (36.7%) of were smokers and (82.8%) had a family member with breast cancer. These results suggested a significant correlation between smoking, family history of cancer, marital status, and breast cancer awareness.

**Conclusion:**

This study showed which factors are significantly related to women’s awareness of breast cancer risk factors. These results can contribute in the implementation of awareness programs and campaigns, thus raising the level of awareness among women.

## Introduction

Highlights

- Breast cancer constitutes the most common malignancy in women.
- Our study suggested a significant correlation between sociodemographic factors, smoking, family history of cancer and breast cancer awareness.
- Our study did not suggest any significant correlation between the level of awareness and residence setting or family history of breast cancer.
- More programs and campaigns about breast cancer should be carried out.

Breast cancer constitutes the most common malignancy in women (38.5%) with an incidence rate of ~46.3 per 100 000, and the second leading cause of cancer-related mortalities (13.8%) with a mortality rate of 13.4 per 100 000. In developing countries, breast cancer has emerged as a major public health concern with a significant increase in incidence rates, and concerning studies mentioned that by 2035 two-thirds of breast cancer cases will be distributed in developing countries^1,2^. In Syria, the Globocan Cancer Observatory reported 4388 new cases in 2020, comprising 20.9% of all cancer cases, with 1964 new deaths^3^. Also, it has been well demonstrated that the 5-year survival rate in developing countries is significantly lower than in developed countries. Multiple factors contribute to the poor prognosis of breast cancer in Syria and other developing countries, and studies revealed that limited resources and late detection represent the major predominant factors^1^.

The healthcare system in Syria has been remarkably affected by the ongoing crisis. The destruction of healthcare facilities, shortage of drugs, reduction of the number of oncology specialists, as well as the disparities in quality of management between major and minor cities have all exacerbated the limitations in oncology care services and decreased prioritization of cancer cases compared to war-related injuries and epidemic diseases including COVID-19^4,5^.

Another major obstacle in our country as well as in other developing countries is late diagnosis. While numerous studies recommend early diagnosis and detection of breast cancer due to its critical role in reducing morbidity and mortality rates, however, these recommendations could not be well-applied in our country due to women’s suspicion provoked by cultural, social, and religious beliefs in addition to economic obstacles^6,7^. Many campaigns are routinely organized by the WHO and national associations in our country to encourage and promote breast cancer routine screening and enhance females’ knowledge of breast cancer risk factors, diagnosis, protection, and self-examination. Nevertheless, studies measuring public awareness are still limited in our country, and most of them concentrate on university students and females in medical colleges mainly in the capital city Damascus^5,8^.

Interestingly, while Tishreen University Hospital which is located in the Syrian coast contains the second-largest Syrian centre for cancer treatment—the largest centre is located at Al-Bairouni Hospital in Damascus^9^, we could not find any study measuring breast cancer awareness in the Syrian coast region. Therefore, we aimed to conduct the first research that measures breast cancer awareness among females residing in urban and rural regions in the Syrian coast.

## Methods

This is a cross-sectional study that was conducted between July and November 2021 and included female residents in the Syrian coast. Data collection was built based on the Breast Cancer Awareness Measurement survey (BCAM) which has been used in several studies, and we modified our questionnaire according to it^10^. Our survey consisted of 38 questions in addition to the consent question at the beginning and questions related to demographic information. Questions related to women’s awareness were closed questions according to the ‘Likert Triangular Scale’. Subsequently, we calculated the arithmetic mean of the questionnaire (2.5173) and its standard deviation (0.177).

A sample size of at least 384 responses was considered adequate in order to have a confidence level of 95% and a confidence interval of 5%. Our target population included females aged greater than or equal to 18 years residing in the Syrian coast’s governorates with an internet access. Males and participants from other regions were excluded. Subsequently, our study sample consisted of 1305 female participants categorized into 1020 and 285 participants from Lattakia governorate Tartous governorate subsequently.

Ethical approval was obtained from the ethical committee at Tishreen University and Tishreen University Hospital prior to the study. Furthermore, consents for participation were obtained individually from each participant.

The statistical package for social science (SPSS version 26) was used for data analysis. The independent samples *t*-test and the one-way analysis of variance (ANOVA) were used to investigate the mean awareness and the relationship between independent variables. *P* value less than 0.05 was considered significant. Our work has been reported in line with the STROCSS criteria^11^ and Standards for Reporting Qualitative Research^12^.

## Results

After excluding male participants and participations from other regions, the studied sample consisted of 1305 forms, divided into 1020 forms from Lattakia and 285 forms from Tartous. Questions related to women’s awareness were closed questions, and questions were ranked according to the ‘Likert Triangular Scale’ as follows (Table 1).

**Table 1:** Questions related to women's awareness were closed questions, and questions were ranked according to the ‘Likert Triangular Scale’ as follows

The answer	I don’t know	No	Yes
Rank	2	1	3

The arithmetic mean of the questionnaire was 2.5173 and standard deviation was 0.177. Over half of the participants (58.5%) aged between 18 and 25. 76.6% of the participants study in university, 40.3% do not work, 74.25% lived in the city and 74.3% were single (Table 2).

**Table 2:** Sociodemographic information of the participants

	Variables	Frequency (1305) Percent (100%)
Age	<18 years	22 (1.7)
	18–25 years	763 (58.5)
	25–40 years	336 (25.7)
	40–60 years	175 (13.4)
	>60 years	9 (0.70)
Highest level of education	I did not complete my education	11 (0.80)
	Elementary school	27 (2.1)
	High school	73 (5.6)
	Intermediate Institute	38 (2.9)
	University	1000 (76.6)
	Postgraduate	156 (12)
Occupation	I do not work	527 (40.3)
	Teaching and academic field	186 (14.3)
	Health field	417 (32)
	Industrial field	33 (2.5)
	Agricultural field	19 (1.5)
	Freelance work	123 (9.4)
Place of living	Countryside	336 (25.75)
	City	969 (74.25)
Governorate of residence	Lattakia	1020 (78.2)
	Tartous	285 (21.8)
Marital status	Single	969 (74.3)
	Married	336 (25.7)

From Table 3, we notice that 36.7% were smokers, 10% of the participants had chronic diseases, 23.1% took certain medications, 82.8% had a family history of breast cancer and 42.2% had one of their family members died of cancer.

**Table 3:** Medical and family history of the participants

Questions		Frequency (1305) Percent (100%)
Do you have chronic diseases?	No	1175 (90)
	Yes	130 (10)
Do you take medications?	No	1003 (76.9)
	Yes	302 (23.1)
Have you or any of your family members had any type of cancer?	No	1284 (98.4)
	Yes	21 (1.6)
Has anyone in your family had breast cancer?	No	1080 (82.8)
	Yes	225 (17.2)
Has any of your family members or relatives had cancer?	No	536 (41.1)
	Yes	769 (58.9)
Has anyone in your family died of cancer?	No	754 (57.8)
	Yes	551 (42.2)
Do you smoke?	No	826 (63.3)
	Yes	479 (36.7)

From Table 4, out of the total participants, we noticed that 28.8% obtained information related to breast cancer from internet, 47.3% believed that females aged between 18 and 50 years old were the most likely to have breast cancer, whereas 47.8% thought that only females older than 40 years old should undergo periodic mammogram. Notably, 46.8% of participants believed that fear of side effects of treatment including hair loss was the most common cause of treatment rejection by patients.

**Table 4:** Questions related to breast cancer knowledge

Variable		Frequency (1305) Percent (100%)
Please tell us what sources do you use to obtain information about breast cancer?	Internet (websites+social media)	376 (28.8)
	Medical journals	80 (6.1)
	Your doctor	111 (8.5)
	Media (TV+radio+awareness campaigns)	145 (11.1)
	Relatives and friends	43 (3.3)
	All previous sources	550 (42.1)
What age group do you think is most likely to have breast cancer?	No specific age	274 (21)
	Less than 18 years old	1 (0.1)
	18–50 years old	617 (47.3)
	More than 50 years old	413 (31.6)
What’s the most common cause to reject complete mastectomy according to your opinion?	The fear of entering the operation room	16 (1.2)
	The fear of long-term consequences of surgery	96 (7.4)
	The fear of others’ opinion	313 (24.0)
	All previous causes	880 (67.4)
What’s the most common cause to reject chemotherapy and radiotherapy according to your opinion?	Thinking that alternative or traditional medicine and herbal complements are better solutions	19 (1.5)
	The fear of side effects like hair loss	611 (46.8)

TV, television.

### Hypothesis testing

As the sample size was large ( n=1305≥30 ), we applied parametric tests. To figure out if the mean of individuals’ answers was bigger than neutrality degree (2) contrary to the alternative “not sure”, we conducted one sample *t*-test as following: Table 5.

**Table 5:** We conducted one sample *t*-test as following

	Test value=2					
					95% CI of the difference	
	t	df	Significance (two-tailed)	Mean difference	Lower	Upper
Mean	105.172	1304	0.000	0.51733	0.5077	0.5270

From Table 5 we found that test index value is 105.172 and its significance probability Sig=0 less than α=0.05. Hence, we reject the null hypothesis and accept the alternative hypothesis indicating that the mean’s value is different from neutrality value 2, and it is bigger than it substantially. As these results also confirm the signal non-parametric test which indicates that answers tend to highlight the existence of breast cancer awareness in women living in the Syrian coast.

### Studying the relationship between females’ awareness towards breast cancer and study variables using Student independent samples *t*-test. **Table 6**

**Table 6:** Relationship results using student independent sample *t*-test

Sentence	t	Significance (two-tailed)	Result
Having chronic diseases	0.085	0.933	(No relation)
Taking medications	−0.417	0.677	(No relation)
Positive family history of breast cancer	−1.247	0.212	(No relation)
Positive family history of cancer	−2.187	0.029	(There is relation)
Death history of cancer	−1.026	0.305	(No relation)
City of residence	−0.817	0.414	(No relation)
Urbanization (the living place rural or urban)	−1.244	0.214	(No relation)
Marital status (married-single)	5.259	0	(There is relation)
Smoking	5.208	0	(There is relation)
Relationship results using One-way ANOVA			
Sentence	F	Significance (two-tailed)	Result
Education level	10.184	0	(There is relation)
Age	6.219	0	(There is relation)
Work	26.733	0	(There is relation)
Sources of information	19.702	0	(There is relation)

Our analysis indicated that there is a statistically significant relationship between women’s awareness towards breast cancer and a positive family history of cancer where the test index value is (−2.187) and its significance Sig=0.029 which is less than α=0.05. Similarly, a significant relationship was found between women’s awareness towards breast cancer and marital status where the test index value is (5.259) and its significance Sig=0. Moreover, breast cancer awareness was strongly associated with smoking as the test index value is (5.208) and its significance Sig=0. Table 6.

To study the relationship between women’s awareness towards breast cancer and some study variable (age, work, education level, and sources of information) we used one-way ANOVA Table 6.

Our analysis suggests that there is a relationship between women’s awareness towards breast cancer and all the aforementioned factors.

## Discussion

Despite the advancement of medical investigations and imaging modalities, high awareness towards breast cancer remains a crucial element in the early diagnosis and detection of breast cancer mainly in low and middle-income countries. In this sense, we conducted the first study in the Syrian coast, where the second-largest Syrian oncology centre receives an increasing number of breast cancer patients^9^. Therefore, investigating females’ awareness towards breast cancer in this region is highly essential.

Our study indicates that breast cancer awareness is fairly associated with age and educational status. The vast majority of our participants aged between 18 and 40 years (with 58.5% are under 25 years) have a university or postgraduate education (88.6%). This group of the population has the best ability to reach internet content and read scientific papers, and as we found in Table 4, this was the most frequent source for obtaining their information. However, we suggest that the high level of education in this group facilitates seeking medical help, so they are the most responding part of the community to breast cancer awareness and early detection campaigns in Syria. Many studies support our findings which demonstrate that high awareness is confined to younger women^13,14^. Another study from China revealed similar results regarding the association between breast cancer awareness and demographic information^15^, which indicates the importance of concentrating on elderly populations in awareness campaigns and screening programs.

On the other hand, the findings of our study did not suggest any significant correlation between the level of awareness and residence setting (Urban/Rural). This result is not consistent with the 41 studies conducted in several countries, which found rural-urban differences in the breast cancer experience^16^. This might be due to attempts to promote health awareness among the rural population and to provide them with healthcare centres, or that urbanization of cities as a result of rural exodus due to the Syrian conflict has led to social, economic, and educational mixing. However, extensive demographic studies are required in Syria to examine this factor. Moreover, having a chronic disease, and taking medications were not associated with the level of awareness, as education about a specific disease was not equivalent to education about breast cancer. And to our knowledge, there is no proof in previous research about a similar relation. This suggests that providing women with curricula discussing health-related topics such as breast cancer in hospitals, clinics and pharmacies could be an effective strategy to raise awareness.

It is also important to highlight the association between breast cancer awareness and both ‘marital’ and employment status. The present study indicates that employed women have good knowledge of breast cancer compared to the unemployed ones. The employment percentage was 59.7% and distributes as following (Teaching and academic field 14.3%, Health field 32%, Industrial field 2.5%, Agriculture field 1.5%, Freelance work 9.4%). Our analysis demonstrated a positive association between employment status and breast cancer awareness. This finding is similar to other papers^15,17,18^. On the other hand, we must highlight that approximately one-third of females in our study work in the health field, in which their job requires health knowledge and enables accessing relevant resources. Therefore, more programs and campaigns should be carried out to raise the awareness level of breast cancer among all society groups.

Furthermore, a causative relationship was found between smoking and breast cancer with ~30% increased risk in active smokers compared to non-smokers according to literature^19,20^. Smoking was associated with a poorer prognosis and higher recurrence rate in breast cancer patients^21^. Interestingly, our study found a statistically significant relationship between breast cancer awareness and smoking (*P* value<0.05), stating that smoker women tend to have less knowledge about breast cancer risk compared to non-smokers. Our result correlates with the study from China^15^, which demonstrated that smoking was independently correlated with breast cancer awareness. In 2010, a study included 46 young participants with 24% of them were smokers, detected low knowledge about the carcinogenic role of tobacco on the breast tissue, although most of them were aware of its role in lung cancer^22^. In 2014, Bottorff *et al*.^23^ found an urgent need to raise the level of awareness about breast cancer risks linked to both active and second-hand smoking among young men and women.

Regarding the medical history, only 1.6% of participants had a personal history of breast cancer. Also, 17.2% of participants reported a positive breast cancer family history. These values are higher than a study conducted by (Ashareef, Basem *et al*.)^24^. Our analysis did not establish a significant association between personal history or family history of breast cancer and breast cancer awareness. This finding is not in line with previous studies^25–27^, which demonstrated that women with a personal or family history of breast cancer were more aware of breast cancer risk factors and early signs. The lack of awareness in our study could be attributable to dependence on the internet and social media as the main information source (28.8%) which could contain false information about breast cancer. In comparison, our study found a statistically significant association between breast cancer awareness and family history of cancer in general (*P*<0.05), indicating that women with a family history of cancer were more aware of breast cancer risk comparing with those who had no family history of cancer. According to literature, most studies regarding the link between family history of cancer and breast cancer awareness focused on breast and ovarian cancer with no other types^28–31^. However, a study performed by Haber *et al*.^32^ found an association between breast cancer awareness and family history in addition to its association with breast cancer family history.

### Limitations

The elements of limitation recognized within this study include: (1) Most of the participants in the study were from the age group (18–40), and this may affect the quality of the results and reduce the impact of other age groups on the study. (2) Most of the participants had a high level of education, and this affects the results because it reduces the impact of the uneducated groups on the study. (3) Most of the participants live in the city, and this affects the results due to the difference in awareness between the city and the countryside.

## Conclusion

In conclusion, our study suggested a significant correlation between sociodemographic factors, smoking, family history of cancer and breast cancer awareness; however, it did not establish a strong association with the residence or family history of breast cancer. Based on these results, more programs and campaigns about breast cancer should be carried out in the most effective ways so accurate information reaches all society groups.

## Ethical approval

We obtained an ethical approval from Tishreen University and Tishreen University Hospital.

## Consent

We obtained individual consent from each eligible female who met the inclusion and exclusion criteria before filling out the questionnaire with informing her of her right to contribute to or withdraw from the study at any time.

## Source of funding

None.

## Author contributions

S.A., H.K., S.I., A.A., J.Z., I.A., M.H., S.J., T.T., Y.H., K.L., S.A., J.S., and S.M. collected the data and participated in drafting and revising the article. M.G. is a professor of oncology and participated in revising the article. O.T. is the biostatistician, analyzed the data. Z.A. is a professor of pathology, the mentor and the guarantor, and critically revised the article.

## Conflicts of interest disclosure

NA.

## Research registration unique identifying number (UIN)

Not applicable.

## Guarantor

Dr Zuheir Alshehabi.

## Availability of data and materials

Data and material are available on reasonable request from the guarantor and mentor of this study Prof. Zuheir Alshehabi.

## Provenance and peer review

Not commissioned, externally peer-reviewed.
